# Agenda-setting in nascent policy subsystems: issue and instrument priorities across venues

**DOI:** 10.1007/s11077-023-09514-5

**Published:** 2023-10-21

**Authors:** Nicole Lemke, Philipp Trein, Frédéric Varone

**Affiliations:** 1 University of Lausanne, Bâtiment Géopolis, 1015 Lausanne, Switzerland; 2 University of Lausanne, Lausanne, Switzerland; 3 University of Geneva, Geneva, Switzerland

**Keywords:** Nascent policy subsystem, Policy instruments, Policy issues, Policy agendas, Institutional venue, Network analysis, Artificial intelligence

## Abstract

The “policy subsystem” has long been a key concept in our understanding of how policies on a given topic are produced. However, we know much less about policymaking in nascent policy subsystems. This article draws on the theories of agenda-setting and venue shopping to argue that the similarity and convergence of policy subsystems’ agendas across different institutional venues and over time are features that distinguish more nascent policy subsystems from their more established, mature counterparts. In simple terms, policy venues’ agendas converge when policy actors begin to discuss the same issues and instruments instead of talking past one another. The article illustrates this argument using textual data on Germany’s emerging Artificial Intelligence (AI) policy: print media debates, parliamentary debates, and a government consultation from the period between November 2017 and November 2019. The insights from our analysis show that actors emphasize somewhat different policy issues and instruments related to AI in different venues. Nevertheless, the longitudinal analysis suggests that the debate does seem to converge across different venues, which indicates the formation of a subsystem-specific policy agenda regarding AI.

**Supplementary Information:**

The online version contains supplementary material available at 10.1007/s11077-023-09514-5.

## Introduction

The concept of the policy subsystem has long been a central element of several established theories of the policy process (McGee & Jones, 2019), such as the Multiple Streams Framework (Kingdon, 2011; Herweg et al. 2018), the Advocacy Coalition Framework (Weible et al., 2011), or the Punctuated Equilibrium Theory (Baumgartner & Jones, 1993, 2002; Jones & Baumgartner, 2005). Policy subsystems are defined as *“networks that actors form or operate within, to interact and coordinate actions with each other to influence the design of policy solutions”* (Ingold et al., 2017, p. 443). Subsystems focus on the policymaking activities around a specific issue on the policy agenda (McGee & Jones, 2019). The concept acknowledges that different policy issues produce specific political dynamics (Lowi, 1972). A subsystem’s boundaries are generally set by geography and/or a particular government level (Weible et al., 2020).

Current research mostly focuses on policy subsystems around issues that have consistently drawn policymakers’ attention for a decade or longer. These subsystems are considered “mature” and generally consist of *“entrenched integrated actors anchored by specialized organizations, subunits of government, and existing policies”* (Nohrstedt & Weible, 2010, p. 9). However, the issues that mobilize such mature subsystems likely represent only a subset of all issues on policymakers’ radar. Thus far, the questions of how such subsystems emerge, develop or fail have received relatively little scholarly attention.

Over the past decade, several scholars have started to address this gap by studying the policy subsystems around emerging issues. This strand of research mostly applies the Advocacy Coalition Framework (ACF) to these “nascent” (as opposed to fully developed and “mature”) policy subsystems (Bandelow & Kundolf, 2011; Beverwijk et al., 2008; Fidelman et al., 2014; Gronow et al., 2021; Ingold et al., 2017; Nohrstedt & Olofsson, 2016; Stritch, 2015), which can be understood as *“issue areas that have only recently emerged on the public agenda, which have little history of public policy outputs, which have previously received little or no serious consideration in public decision-making forums, and where advocates have only recently become active”* (Stritch, 2015, p. 438). The findings show that policymaking in emerging issue areas, i.e., nascent policy subsystems, is distinct from policymaking in more mature, established policy subsystems (Ingold et al., 2017).

This conclusion demonstrates that the current focus on mature policy subsystems neglects more dynamic aspects of policymaking, such as how new issues and policy solutions are framed and reach decision-makers’ agendas. Focusing on nascent subsystems around emerging issues thus offers a new perspective on agenda-setting, the preconditions for policy change, and policymaking processes that do not take place in closed advocacy coalitions or policy communities (Ingold et al., 2016). Furthermore, it allows us to account for policy issues that are on policymakers’ agenda but around which fully mature policy subsystems might never develop, and to identify the factors that affect the development of these issues. We thus ameliorate the survivalist bias (Tilly, 1975) of the current approach to policy subsystems, which mainly focuses on those subsystems that have successfully reached a mature stage (which are most likely to be exceptions rather than the general rule). A central advantage of this novel perspective is that it helps us to better understand if and how very recent policy issues, such as climate change and artificial intelligence, are integrated into existing policy subsystems, or if they lead to the formation of new communities of policymakers (Cejudo & Trein, 2023).

This article contributes to the literature on policy subsystems in two main ways. First, we connect the concept of the nascent policy subsystem to the fields of agenda-setting and venue shopping. We use the literature on agenda-setting and, more specifically, on the punctuated equilibrium theory (PET) as a toolkit to develop a description of agenda-setting processes in nascent policy subsystems. We do not seek to test the PET’s power to explain policy change in nascent policy subsystems, but rather use some of its elements to shed light on the differences between nascent (emerging) and mature (established) policy subsystems. We argue that analyzing these patterns can help us understand where a policy subsystem is located on the nascency-maturity continuum. Specifically, we focus on how actors link the topic in an emerging policy subsystem to established policy issues and policy instruments in different venues at the domestic level. We argue that (a) nascent policy subsystems differ from their more mature counterparts in that they exhibit a diversity of policy issues and instruments across venues and (b) the maturation of policy subsystems occurs when issues and instruments converge across venues.

Empirically, this argument suggests that nascent policy subsystems should exhibit diversity in the links between their central policy issues 1 and policy instruments^2^ across different policymaking venues.^3^ If a nascent policy subsystem develops toward maturity, the link between issues and instruments converges in different venues over time. In other words, a policy subsystem's agenda converges because policy actors begin to discuss the same issues and instruments related to the topic at hand instead of talking past one another. In contrast, whether they agree or disagree on these issues and instruments is important but a separate question altogether.

Second, we strive to make a methodological contribution to the literature on nascent policy subsystems. We contend that discourse network analysis (DNA) is a fitting tool for the analysis of nascent policy subsystems’ degree of maturity. DNA is a special form of social network analysis that emphasizes policy debates’ networked character (Leifeld, 2017). It also allows us to efficiently examine how actors link policy issues and policy instruments in different venues. We would like to emphasize that we only use DNA as a method of analysis and do not theorize the role discourses and narratives play in policy in nascent policy subsystems (Jones et al., 2023).

We use the case of Germany’s policy on artificial intelligence (AI) between 2017 and 2019 to illustrate our claims. Governing AI is a challenging task (Taeihagh, 2021), and governments all over the world have started to produce policies on the topic (Fatima et al., 2020; Guenduez & Mettler, 2022; Radu, 2021; Ulnicane et al., 2022). Germany is no exception and adopted a national AI strategy in 2018 (OECD.AI, 2021), which can be considered a first indicator of a potentially developing subsystem on AI policy. Our analysis is based on an original dataset of 1,035 statements on AI made by representatives of political parties, interest groups, scientific experts, and public officials in parliamentary debates, a government consultation, and print media debates between 2017 and 2019, i.e., one year before and one year after the adoption of the German strategy on AI.

Our case illustrates that the issue and instrument priorities and linkages in policy debates across different venues generally present the German subsystem on AI policy in the period between 2017 and 2019 as more nascent than mature. This is consistent with what we would expect from a subsystem forming around an emerging topic, such as AI. Nevertheless, there are some signs that the agendas of the actors debating the topic in different venues started to converge between 2017 and 2019, even though we focus on a very short period. At the national level, it remains to be seen whether Germany will develop a policy subsystem on AI or if existing policy communities will be in charge of AI.

## Issue and instrument priorities across venues

Previous research on nascent policy subsystems mostly follows the tradition of the ACF, which identifies subsystems either via the actors they involve (e.g., advocacy coalitions) or via their substantive content (e.g., belief systems). So far, scholars have mainly focused on how actors' coalitions and their subsystem-specific core policy beliefs in nascent contexts differ from what we know about their counterparts in established policy subsystems. This stream of research indicates that the logic policymaking follows in nascent subsystems is different from that in mature subsystems: Most of the former are characterized by a rather collaborative appearance (Fidelman et al., 2014; Ingold et al., 2017; Nohrstedt & Olofsson, 2016). Thus, actors’ alliances often do not yet resemble the clear-cut advocacy coalitions typical of mature subsystems (Weible et al., 2020). Similarly, their agendas and issue-specific beliefs are not well defined (Ingold et al., 2017; Nohrstedt & Olofsson, 2016; Stritch, 2015). Actors in nascent policy subsystems thus find it difficult to identify peers who share similar ideologies and rely on former contacts to form alliances more often than in mature subsystems (Ingold et al., 2017).

Even though our understanding of the differences between nascent and mature policy subsystems has greatly advanced thanks to the ACF-driven research, we still lack a conceptualization of “nascency” that would allow us to locate the policymaking activities around a particular issue on a scale between a “nascent” (or emerging) subsystem and the ideal type of a more stable, “mature” policy subsystem with specialized actors, integrated (advocacy) coalitions, and dedicated government institutions (such as a regulatory agency). Most of the literature on nascent policy subsystems assumes that a policy subsystem is nascent because it tackles an issue that is rather new to the policymaking process (such as emerging technologies) and thus has only existed for less than a decade—the typical minimum life of most subsystems studied by the ACF (Sabatier & Jenkins-Smith, 1999). This assumption of nascency is rarely put to a test.

However, we need to be able to empirically place a policy subsystem on the nascency-maturity scale in order to fully grasp how policymaking in nascent contexts compares to policymaking in established policy subsystems. Temporal development is not a reliable indicator on its own, because it neglects that some nascent policy subsystems can develop mature features over relatively short periods of time (Stritch, 2015), while others may display nascent characteristics for a long time after their creation (Beverwijk et al., 2008). Additionally, established policy subsystems in the middle of punctuations of policy change might briefly exhibit empirical features similar to those of nascent policy subsystems (such as instability in the coalitions of relevant actors) before settling into new and stable equilibria. Moreover, some of the “nascent” characteristics identified by previous research overlap with other attributes that even policy subsystems that our definition considers mature may vary on. Collaborative appearance is one such attribute. Although it seems to be present in many of the subsystems previous research has identified as nascent (Fidelman et al., 2014; Ingold et al., 2017; Nohrstedt & Olofsson, 2016), it also appears in policy subsystems that have been classified as mature (Weible, 2008).

To ensure that future research on nascent policy subsystems does not suffer from the same arbitrariness that plagues the current definition of mature subsystems, we therefore need to clearly define and conceptualize a set of characteristics that would allow us to place policymaking on a specific topic along the nascency-maturity continuum. Existing research suggests that both the role of policy actors’ interactions and the policy beliefs the ACF has examined are fruitful avenues for further research.

In this article, we seek to fill the existing gap by putting forth the attention on and prioritization of policy issues as a promising measure of a policy subsystem’s degree of nascency or maturity. Our theoretical argument is twofold: First, we argue that the emergence of a new policy subsystem generates unique incentives for policy actors’ strategic behavior that in turn affect how they raise policy issues and policy instruments in different venues. Second, we proceed to argue that the incentives for such strategic behavior shift as the policy subsystem gradually gains maturity, leading to the development of a unique agenda of policy issues and instruments across the policy subsystem, which we can use to place the latter along a nascency-maturity continuum.

### Issues, instruments, and venues in nascent policy subsystems

Policymakers produce policies on relatively new issues all the time (for the case of AI, see OECD.AI, 2021). This means that actors must engage on various policy issues (such as the policy problems raised by the new subsystem’s central topic) and propose policy solutions (including policy instruments) even in presumably nascent policy subsystems. The literature on agenda-setting demonstrates that the choice of issues that should receive political attention takes place in the early stages of the political process and that this choice is highly relevant to subsequent political action (Green-Pedersen & Walgrave, 2014). Policy actors, such as state agencies, political parties, interest groups, academic experts, etc., are constrained by limited time and resources and prioritize certain issues over others. They strive to put these issues on the political agenda.

We define *policy issues* as the public problems that actors who belong to an existing policy subsystem identify as important priorities in need of addressing by new public policies. This definition allows us to adopt a broad perspective on the policy process that transcends the belief system’s structure. The latter has been suggested by ACF proponents and is difficult to empirically grasp in nascent subsystems. During the agenda-setting phase, actors strategically link certain issues to further their agenda (Tosun & Varone, 2021). Moreover, actors consider policy issues (or policy problems) and related policy solutions simultaneously (Baumgartner & Jones, 2015; Kingdon, 1993).

We define these solutions as *policy instruments*, i.e., the tools actors use to reach politically defined objectives on the subsystem’s agenda. Policy instruments are the element that connects all remaining elements of the policy design—the policy objectives, the implementation arrangement, and the target groups (Howlett & Lejano, 2013; Landry & Varone, 2005; Linder & Peters, 1989; Schneider & Ingram, 1993). Policy instruments thus act as the policy design’s “glue” because they are used to achieve policy goals, get implemented by public agencies, and work to modify target groups’ behavior. Therefore, it is not surprising that several studies that draw on the ACF have also used actors’ support for the same policy instrument to identify the members of an advocacy coalition (Fischer, 2014; Ingold, 2011; Ingold & Varone, 2012).

Regarding solutions, actors look for policy instruments that they consider better suited to tackle the problem related to the emerging policy subsystem (Linder & Peters, 1989; see Howlett, 2023 for an overview). Notably, scholars have argued that policy actors are organized in instrument constituencies (i.e., around specific policy instruments) and tend to support specific policy solutions (e.g., regulations or financial investments) once a new policy problem appears (Béland et al., 2018; Linder & Peters, 1984; Weible, 2018). In other words, issues and instruments float around in the “policy primeval soup” waiting for actors to connect them (Kingdon, 2011).

Policy instruments can include bans, prescriptions, taxes, subsidies, standards, information campaigns, and voluntary measures. Most often, policy instruments fall into one of three categories (Vedung, 1998): regulative (sticks), incentive-based (carrots), and persuasive measures (sermons). Each successive type in this sequence entails a lower degree of coercion on the target group and a reduced intensity of the state intervention. Furthermore, policy instruments can also seek to increase actors’ cooperation in the policy process, such as, for example, between different ministries, levels of government, or European Union member states (Howlett, 2000; Trein & Ansell, 2020). Alternatively, actors may decide that private self-regulation is preferable to any state intervention. Therefore, self-regulation, such as, for example, that by non-state actors, is another category of instruments that actors can put on a nascent policy subsystem’s agenda (Hirsch, 2011; Pattberg, 2005).

The set of policy issues and policy instruments that makes it onto a subsystem’s agenda is not arbitrary. Different policy issues always compete to get onto the subsystem’s policy agenda, because policy actors' attention is a very scarce resource. Different *political venues* are a key part of this agenda-setting process. Actors engage in venue shopping to maximize their agenda-setting power when they try to put their preferred policy issues and instruments on a policy subsystem’s agenda (Baumgartner & Jones, 1993; Jourdain et al., 2017; Pralle, 2003). Taken together, different venues are accessible to different types of actors with potentially diverging issue priorities and instrument preferences. They also offer those actors different opportunity structures.

### Convergence of issue and instrument priorities across venues

Against this background, we expect a high divergence of issues and instruments across different policymaking venues in a nascent policy subsystem. In other words, the various actors define the problem and the solution in different terms across media debates, parliamentary debates, interest group consultations, and other venues. This divergence should be particularly pronounced when the topic has only recently emerged, as is the case in nascent policy subsystems. Meanwhile, the policy agenda should be more clearly defined in mature subsystems. We can capitalize on this difference to estimate policy subsystems’ degree of nascency or maturity by analyzing and comparing the policy issues and instruments that are prioritized and linked across a subsystem’s venues. We define convergence as a policy subsystem’s development from a diverse agenda of issues and instruments to a clearly defined, subsystem-wide agenda of similar issues and instruments across venues. In the following paragraphs, we explain why we develop this expectation based on the literature on policy agendas.

Agenda convergence takes place because of policy actors’ strategic behavior under the different incentive structures nascent and mature policy subsystems offer. Mature, established policy subsystems are characterized by relatively stable coalitions of actors (Nohrstedt & Weible, 2010). Consequently, those subsystems contain dominant actors who act as gatekeepers and are able to control the framing of policy issues (Baumgartner & Jones, 1993, 2002; Jones & Baumgartner, 2005). The presence of established gatekeepers means that the agenda-setting process likely is less open and competitive in mature policy subsystems than in nascent subsystems.

The following two mechanisms can illustrate the convergence of policy priorities in the transition from a nascent to a mature policy subsystem: (1) The search for expertise and (2) feedback effects. First, Baumgartner and Jones (2015) argue that once governments face new topics, they engage in a process of gathering information, or a “search.” The latter creates a demand for diverse expertise on how to address the issues in the nascent policy subsystem (Baumgartner & Jones, 2015). The degree of uncertainty in different actors’ definitions of the problem and its solution raises the demand for diverse expertise in nascent policy subsystems and lowers it in their mature counterparts. Therefore, instead of engaging with the issues and instruments their peers put on the agenda, policy actors in nascent policy subsystems compete to draw attention to their preferred policy issues and instruments and engage in venue-shopping, which causes a plethora of issues and instruments to appear on the policy subsystem’s agenda.

However, this type of strategic behavior comes at a cost: *“The struggle between information and control affects the ability of policy communities to maintain support and consensus about “best practices” in their respective policy niches”* (Baumgartner & Jones, 2015, p. 61). In other words, as the pressure to prioritize certain problems and solutions increases, the scope of issues and instruments on the nascent policy subsystem’s agenda narrows because actors find it harder to defend their own issues and instruments and are more hard pressed to engage with other actors’ issues and instruments. Consequently, actors form communities around specific problem definitions and solutions in a dominant policy arena, which indicates that a policy subsystem is mature (Kammerer & Ingold, 2021).

Second, positive feedback effects should lead to a convergence of the issue-instrument links and priorities on a subsystem’s agenda. The appearance of such a set of similar issue and instrument links and priorities across venues within a policy subsystem can therefore be seen as an indicator of a policy subsystem’s maturity. Positive feedback effects facilitate the quick absorption of external shocks through policy change, whereas negative feedback effects constitute reactions that maintain the subsystem’s stability against external disturbances (Jacobs & Weaver, 2015, p. 443). The negative feedback effects should be weaker in nascent policy subsystems than in mature policy subsystems, because the latter are more stable. If a nascent policy subsystem gains maturity, the convergence of the policy subsystem’s agenda could be supported by positive, self-reinforcing feedback effects. Processes such as mimicking (actors imitating one other’s behavior) and attention-shifting (actors being forced to pay attention to issues other actors are pushing) should raise actors’ tendency to refer to the same sets of issues and instruments on a policy subsystem’s agenda across venues (Baumgartner & Jones, 2002). It is important to note that the convergence of a subsystem’s policy agenda is not the same as a policy subsystem without conflict. Policy subsystems that are classified as mature exhibit varying degrees of conflict and consensus (Weible, 2018).

The establishment of a subsystem-specific agenda of issues and instruments does not take place in all emerging issue areas. Therefore, some nascent subsystems might never mature past their early stages of development and collapse or get absorbed by other subsystems, instead (Ingold et al., 2016). Our argument suggests that, in these cases, we would never see the development of a clearly identifiable, subsystem-wide policy agenda. Rather, the issues and instruments policy actors link and prioritize when they address the subsystem’s topic would remain diverse and different venues’ agendas would look different.

Taken together, the studies on agenda setting and policy instruments lead us to formulate the following expectations: In nascent policy subsystems, actors emphasize diverse policy issues and instruments across different venues, which is not the case in mature policy subsystems. Furthermore, as a policy subsystem becomes more mature, this diversity declines over time.

We recognize that these expectations are descriptive but highlight that at the current level of knowledge, descriptive analyses constitute an important step toward the empirical identification of agenda-setting in nascent policy subsystems (Gerring, 2012). The goal of our analysis is to use various theoretical elements related to agenda-setting to provide a better understanding of such dynamics in nascent policy subsystems and not to test different theoretical approaches against one another.

It is important to keep in mind that issue and instrument priorities form in nascent policy subsystems even before stable core policy beliefs emerge and patterns of actor cooperation solidify within advocacy coalitions. In other words, actors refer to, link, and prioritize different policy issues and policy instruments before they begin to build the formal coalitions typical of more mature policy subsystems. This is crucial, because it makes it possible for our analysis to include emerging issue areas that exhibit very nascent characteristics, including areas that might never develop beyond the stage of nascent policy subsystems, as well as established policy subsystems that exhibit more mature characteristics, in which clear actor coalitions and policy beliefs are difficult to identify.

## Towards an empirical analysis: case study and data

Not only does this paper strive to make a conceptual contribution on agenda-setting in nascent policy subsystems, it also proposes a new approach to studying said phenomenon in such contexts. For the purposes of this endeavor, we mobilize established strategies from the comparative research on policy agendas across various venues (Green-Pedersen & Walgrave, 2014). We examine parliamentary debates (Dolezal et al., 2014), a government consultation (Culpepper, 2010), and print media debates (Esser & Strömbäck, 2014; Tresch et al., 2013).

Focusing on textual data from policy debates is not the only way to examine actors’ preferences for issues and instruments. However, this sort of data allows us to simultaneously capture issue preferences, instrument preferences, and the links between issues and instruments, as well as the agenda-setting dynamics involved in actors’ choice of venues. This quality makes it a good choice for the operationalization of the theoretical argument proposed in this article.

To empirically illustrate our argument, we use data from the political debate on AI in Germany and compare information from three venues: a government consultation with interest groups on the topic of Germany’s national strategy on AI, the Federal Parliament, and the media (two quality newspapers, *Frankfurter Allgemeine Zeitung* and *Süddeutsche Zeitung*). We use data from Germany because this country is likely to be a typical case (Gerring, 2016) of a nascent AI policy subsystem: It is the largest member state of the EU and therefore plays a vital role in EU’s policymaking on AI (Justo-Hanani, 2022). Digital issues have started to occupy an important place on the German policy agenda (Beyer et al., 2022). Moreover, like other developed economies and liberal democracies around the world, Germany has developed a national strategy on AI that aspires to serve as a framework for further policy action (Radu, 2021). Germany is lagging behind the frontrunners of AI development and policy, such as China, the UK, and the USA (OECD.AI, 2021), but it does count among the frontrunners in the EU (Justo-Hanani, 2022). It is plausible to assume that Germany’s policy on AI is a typical case of a nascent policy subsystem, given that its subject (AI) constitutes a rapidly developing set of technological innovations which has only recently started to receive broad political attention (OECD.AI, 2021). More generally, it gives us the chance to study the intersection of science and public policy (cf. Tosun, 2017; Tosun & Schaub, 2017).

The German Federal Government has repeatedly identified AI as a policy problem of central importance to Germany's and Europe's future competitiveness and places in the world.^4^ Therefore, the focal point of our analysis is Germany’s national strategy on AI (*KI-Strategie der Bundesregierung*). Created in 2018, it aims to develop a coherent AI policy, and therefore is a useful starting point to explore the emerging issue area around AI in Germany.^5^ We use textual data from policy debates in three different venues. We collected data for the period between November 2017 and November 2019, a year before and after the implementation of the German national strategy on AI.

Our original set of documents consists of different types of textual data, which correspond to the debate about AI in the aforementioned policymaking venues. We use a random sample of 281 articles out of a total population of 2,865 newspaper articles printed in two quality newspapers that occupy different positions on the political spectrum–the Frankfurter Allgemeine Zeitung and the Süddeutsche Zeitung. We built our original dataset by searching for the terms “artificial intelligence” and “algorithm” (“Künstliche Intelligenz” and “Algorithm*”). The 281 articles cover the entire period we examine. In each of the selected articles, we coded the direct or indirect statements various actors made on AI. The textual data from the parliamentary venue consist of 356 individual interventions by members of parliament. We collected them from the Federal Parliament’s database by using the same search terms and identifying all relevant debate protocols from the period in question. We proceeded to extract interventions made by members of parliament in debates about AI. Our data set also includes 818 stakeholder comments from the government consultation on the national strategy on AI, which took place in autumn 2018. These comments were delivered in response to a government document and were supposed to follow a predefined structure with open sections. Actors greatly differed in their use of this structure, and many offered a commentary on points that fell outside of its scope. Our analysis considers all raised points because they clearly refer to AI.

Our analysis follows the two-step procedure typical of empirical discourse network analysis (Leifeld, 2017). We first conducted a category-based content analysis. Then, we performed a network analysis on the data set constructed in the first step. The first step consisted in us going through the documents (newspaper articles, policy statements, parliamentary interventions) and coding “statements,” which represent the smallest unit of analysis in DNA (Leifeld, 2017). We applied a “thematic criterion” (Schreier, 2012), which means that the statements’ lengths range from a few words to entire sentences or paragraphs depending on which excerpt of the text best captures the variables of interest. We used Leifeld's (2019) software *Discourse Network Analyzer* to code four variables for each statement: The name and type (organizational or individual) of the actor making the statement, the policy issue they raised in relation to AI, which was coded based on the 21 policy categories suggested in the codebook of the Comparative Agendas Project (cf. Baumgartner et al., 2019), and the policy instrument that the statement referred to, which was coded based on five categories (i.e., non-state action, information and education, cooperation and coordination, investments and incentives, and regulation and legal framework). Table 1 in the Online Appendix shows that the coded statements were made by a diverse set of policy actors who mobilized in different venues.

We manually coded the actors’ statements in two rounds. A first coder assigned policy issue and policy instrument categories to the text. Then a second coder checked these categories to ensure our classification’s reliability. One limitation of this approach is that we were unable to calculate an intercoder reliability score. Prior to analyzing the data, we applied a set of measures meant to control for the inherent biases of our data sources. We excluded instances when the same document referred to the same policy issue multiple times. We also accounted for the institutional biases of the parliamentary and the newspaper venues by employing average activity normalization, which controls for the advantages these venues give some actors, which make them appear more active (Leifeld, 2017; Leifeld et al., 2019). We did not apply the same measure to the consultation because, in theory, it was open to anyone (cf. Kukkonen & Ylä-Anttila, 2020; Leifeld, 2017).

## Methods

To illustrate our theoretical argument, we use a cross-sectional comparison of the debates held in the three different venues. We use descriptive measures of discourse network analysis (DNA) to empirically capture this dimension. DNA is a particular type of social network analysis (SNA) that applies the toolbox of network analysis to political debates (Leifeld, 2017).

Networks are one of the most straightforward ways of conceptualizing policy subsystems (McGee & Jones, 2019). Unsurprisingly, SNA is frequently applied to the analysis of mature policy subsystems, with great success. For example, it is well suited to identify advocacy coalitions and the choice of policy instruments (Ingold & Varone, 2012) and to trace the advocacy activities on a specific issue across venues (Jourdain et al., 2017; Varone et al., 2017a, 2017b). Furthermore, SNA has also already been successfully applied to nascent policy subsystems (Ingold et al., 2017). This literature sheds light on how maturity affects coalition formation in nascent policy subsystems. The existing research thus demonstrates that SNA is well suited to investigate relations among actors, such as trust, cooperation, or power, in both mature and nascent policy subsystems.

In contrast, DNA emphasizes how actors relate to one another in political debates (Leifeld, 2017). DNA conceptualizes political debates as a *“network phenomenon”* because the statements actors make in a political debate are interdependent and therefore *“constitute relational action”* (Leifeld, 2017, p. 1). Hence, one advantage of DNA is that it simultaneously accounts for issues and actors, as well as their interdependence (Leifeld, 2017). It thus allows us 1) to explore how the sets of policy issues and instruments on a policy subsystem’s agenda are connected via the actors who refer to them, and 2) to determine whether their prioritization and the links between them converge across venues, which indicates subsystem maturity. Since it relies on textual data from various sources instead of the survey data SNA typically draws on, DNA holds additional advantages for the analysis of nascent policy subsystems: First, it makes it possible to collect data on any specific time frame ex post. Therefore, it is a useful tool for investigating the multiple stages of subsystem development in retrospect. Second, collecting longitudinal data is less costly than conducting surveys.

Our analysis uses a two-mode network approach because we are interested in more than one set of concepts (issues and instruments) and in how actors link them on the policy agenda (Agneessens & Everett, 2013; Borgatti & Everett, 1997; Brandenberger et al., 2020). In contrast to one-mode networks, two-mode networks consist of two types of nodes and display the connections between nodes of different types, but do not allow for links between nodes of the same type. In our case, network nodes represent the policy issues and policy instruments actors mention in the documents from the government consultation and the parliamentary and newspaper debates. The links between the nodes represent joint mentions of the particular issue and instrument by actors in these documents. To construct the networks, we calculated the co-occurrence matrices of the issue and instrument variables in our dataset, whereby each entry represents the overall count of a particular issue-instrument pair. The data are organized in a way that links statements with actors, issues, and instruments, so they should be interpreted as the number of times an actor links a policy issue and a policy instrument. Link weights between policy issue nodes and policy instrument nodes increase in proportion to the number of times actors mention them jointly. By showing how actors link issues and instruments on the agenda and how strong these links are, our two-mode networks thus allow us to investigate the similarities among the issue-instrument pairs across a subsystem’s venues.

To make the comparison of (potential) similarities more explicit, we rely on the degree centralities of issue and instrument nodes. Degree centrality can be defined as *“the number of ties of a given type a node has*” (Borgatti et al., 2018, p. 191). Typically, the node with the highest degree centrality is the node with the most connections to other nodes. Our measure draws on Leifeld and Haunss’ (2012) indicator of the core frames of a political debate. The authors use the degree centrality of concept nodes in a one-mode network to operationalize the dominant frames in a political debate. In a similar fashion, we use degree centrality to assess which issues (policy problems) and instruments (policy solutions) actors prioritize in debates that take place in different venues. Those can therefore be considered important to the policy agenda. We use a “dual-projection approach” to calculate the degree centralities in the two-node networks (Everett, 2016; Everett & Borgatti, 2013). This approach consists in finding the centralities of both sets of nodes in the projected one-mode networks and then mapping them back onto the two-mode network by considering their interdependence (Everett, 2016). We choose this procedure because we regard issues and instruments as complementary; therefore, issues’ centralities depend on the centralities of the instruments they are linked to, and vice versa.

We acknowledge that the role of time is crucial in analyzing a policy subsystem’s development from nascency to maturity (Kammerer & Ingold, 2021). We therefore also conduct a temporal analysis of the policy debates in newspapers and in the Federal Parliament. The data from the government consultation are not longitudinal in nature and should therefore not be included. For this analysis, we divided the newspaper and parliament data in two time periods – one before and one after the adoption of the national strategy on AI (November 2017 to October 2018 and November 2018 to November 2019) – and applied our method to the respective datasets. We regard our cross-sectional comparison across venues as complementary to the temporal comparison of the networks: An analysis would ideally include both angles. Yet, if a topic is very new to policymaking (as is the case of Germany’s AI policy), data on its temporal development are naturally scarce. The more restricted version of a snippet-like cross-sectional comparison across venues could therefore represent a viable alternative to analyze a subsystem’s degree of nascency or maturity (Ingold et al., 2016).

## Results

### Similarity of policy issues across venues

**Fig. 1:**
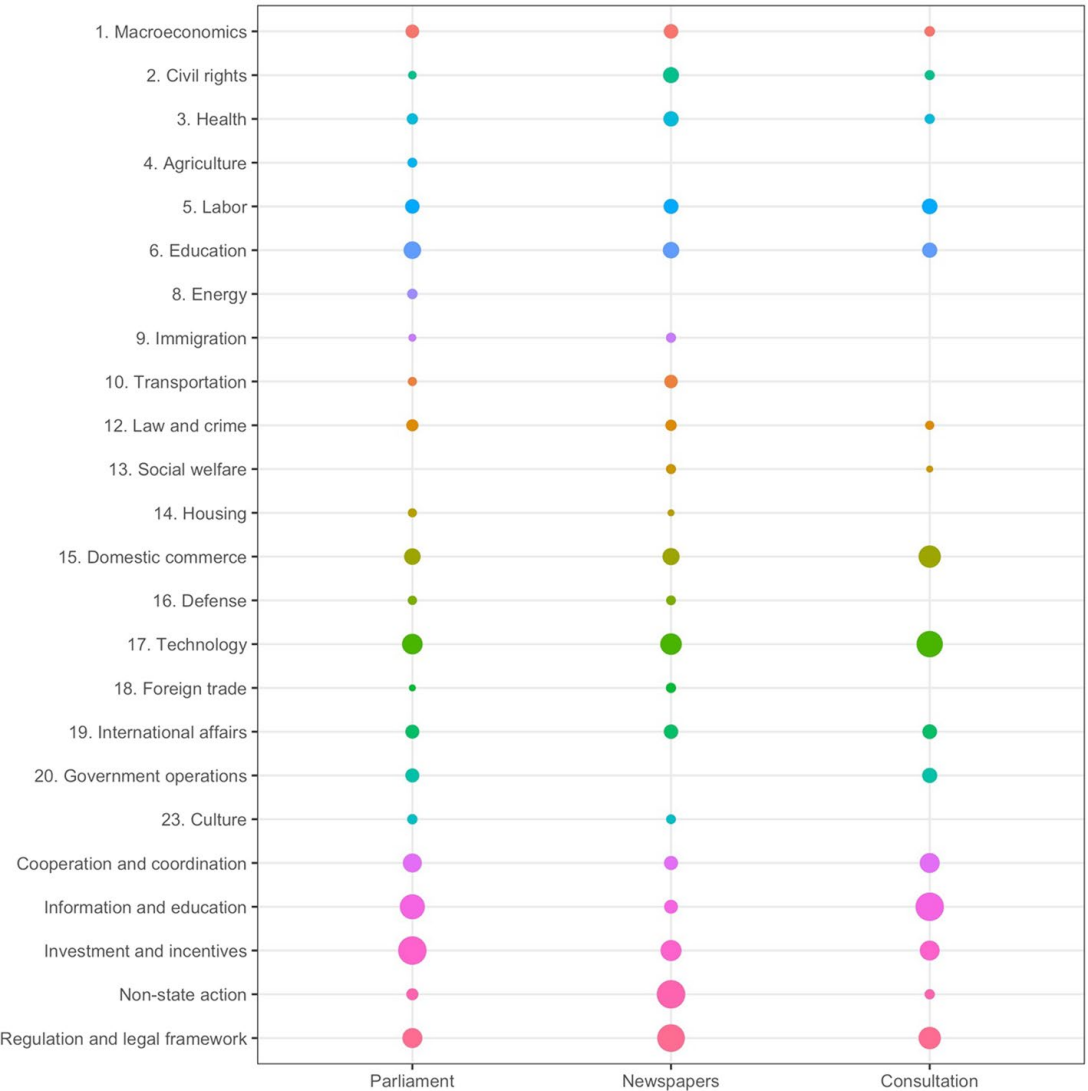
*Issues’ and instruments’ degree centralities across venues.* Degree centralities across venues (the variables are normalized to take on values between 0 and 1): Larger circles represent higher degree centralities. Certain issues do not appear in all venues; hence, the blank spaces. Overall, instrument nodes’ higher degree centralities are related to the smaller number of nodes. Therefore, we refrain from comparing the two different sets of nodes. Colors serve to clearly differentiate the individual lines

Figure 1 presents the results of the degree centrality analysis of the two-mode networks. We used R (version 4.2.2, R Core Team, 2020) to produce the calculations and the ggplot2 package (Wickham, 2016) to visualize them. The comparison of the issues’ and the instruments’ degree centralities across the parliamentary debates, print media, and the government’s consultation with interest groups reveals two main findings. First, even though the degree centralities vary across venues, several policy issues are very central in all three venues. Second, the highest values of degree centrality for policy instruments appear to be rather diverse across venues.

We focus on the five policy issues with the highest degree centrality in each venue. Technology, domestic commerce, and education display consistently high values across the print media, parliamentary debates, and the government consultation. For example, the media cites a big industrial organization, which describes the EU’s regulations on AI as “an important step for AI made in Europe” but also warns the Union to “treat all applications the same way.” We code this as “technology.” Similarly, labor and government operations are important in two of the three venues: the consultation and Parliament. One example of “government operations” from the parliamentary debate is one Christian Democrat MP’s declaration that they “believe that the structures with the Minister of State in the Federal Chancellery, with the Digital Cabinet or the State Secretary Committee, which coordinates, are absolutely the right way to go.” In contrast, civil rights and health have relatively higher values in the media venue. For example, one article indirectly cites a professional medical organization, which warns that it is “important that there is always a contracted physician at the end of” diagnostic apps. We coded this statement as “health.” Additional examples of the statements we coded can be found in Table 2 in the Online Appendix.

Nevertheless, if we extend the analysis beyond the five most central issues, labor also follows closely in the media venue. Since degree centrality can be interpreted as an issue’s prominence or importance in the policy debate, these findings indicate that actors prioritize similar issues across the three venues. Therefore, these results can be interpreted as a first sign of a subsystem-specific policy agenda in the German subsystem on AI policy. It appears that German policy actors have indeed started to talk about a characteristic set of policy problems that matter with respect to AI. Since mature subsystems are characterized by a policy agenda with a fixed and potentially limited set of central policy issues across all venues, this finding suggests that the subsystem is no longer fully nascent, even though it had only emerged shortly before the period of our study.

### Limited similarity in the links between issues and instruments across venues

**Fig. 2:**
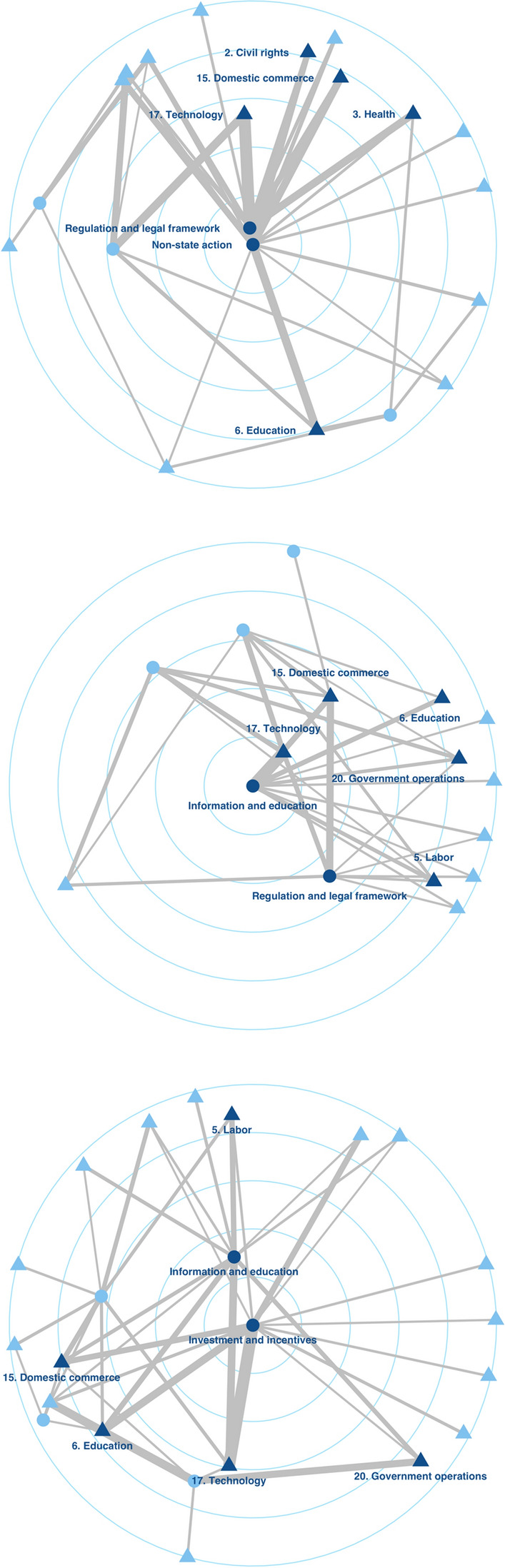
*Policy issue—policy instrument networks across venues (centrality layout)*. Two-mode networks (from top to bottom: media, consultation, parliament): Nodes: Policy issues are depicted as triangles, while policy instruments are depicted as circles. The dark blue color highlights the five issue nodes and the two instrument nodes with the highest degree centralities. Edges: Their width denotes the strength of each edge

Figure 2 presents the two-mode networks of policy issues and policy instruments across venues. We used R (version 4.2.2, R Core Team, 2020) to pre-process the data and the package igraph (Csardi & Nepusz, 2006) to visualize the networks. The latter are organized by their degree centralities, with nodes showing higher values placed closer to the center of the circle. In addition to highlighting the prominence of issues and instruments across venues, the networks also illustrate the connections among them. This makes them a useful operationalization of the “map” of issues and instruments that make up a policy subsystem’s agenda. They allow us to interpret and compare the following three characteristics of the debates taking place in a subsystem’s venues: First, the closer to the center of the circle a policy issue or instrument is, the more central, and thus important, it is in the policy debate. This allows us to compare the relative importance of policy issues across venues. Second, we can compare how similar the number and type of issues and instruments are across the graphs. Third, we can determine the strength of the connections between issues and instruments by analyzing the strength of the network ties.

Just like Figs. 1, 2 shows a lack of similarity in the policy instruments mentioned in the policy debates across our venues: The policy instruments with the highest degree centralities across the three venues are rather diverse. The most central policy instruments in the media debate are non-state action and regulation and legal framework. Information and education and regulation and legal framework are most central in the government consultation. Finally, investment and incentives and information and education are most central in the parliamentary debate. Table 2 in the Appendix lists some coding examples of the different instruments. Similarities do exist between the government consultation on the one hand and the media debate (regulation and legal framework) and the parliamentary debate (information and education) on the other. This finding constitutes a pattern we would expect based on the consultation’s intended goal of connecting policymakers and the broader public. As our comparison shows, overall, the overlap between the most central policy instruments is not clear enough to speak of real similarity, especially in the parliamentary and the media venues.

Moreover, how strongly policy issues are connected to various policy instruments also varies across venues. In the news media, the issue of technology is strongly linked to the instruments of non-state action, regulation and legal framework, as well as investment and incentives. In contrast, in the consultation venue, it is most strongly linked to information and education and less strongly connected to cooperation and coordination and investment and incentives. Finally, the strongest link in the parliamentary venue is that between technology and investment and incentives, followed by the one between information and education. While a certain degree of similarity (e.g., the link to investment and incentives) is visible, the differences across the venues seem to be more pronounced.

Technology is not the only policy issue with this type of mixed results. They are also present in the case of domestic commerce, which is most strongly linked to the instruments of regulation and legal framework and non-state action in the media venue, regulation and legal framework and information and education in the consultation venue, and investment and incentives and regulation and legal framework in the parliamentary venue. Thus, while domestic commerce is relatively strongly linked to the instrument of regulation and legal framework in all three venues, it is not as strongly related to other policy instruments. Another aspect that attests to the differences across the venues is the role of non-state action: While it is strongly linked to a variety of policy issues in the media venue, these links do not exist in the consultation and the parliamentary venues. The links among other issues and instruments can be found in Figure 4 in the Online Appendix.

Our comparison of these debate networks sheds light on concrete combinations of problems and solutions on the agenda of our policymaking venues. A similarity across venues can be considered an indicator of a clearly defined subsystem-wide agenda and, therefore, a more mature policy subsystem. As our results demonstrate, this type of convergence is still rather limited in the case of Germany’s policy on AI during the observed period (2017-2019). Even though policy actors in the print media, the parliament, and the government consultation all somewhat agreed on the most important policy problems for AI, the policy agenda seemingly continues to be more volatile when it comes to identifying concrete policy solutions during the period observed. The lack of similarity across venues indicates that policy actors are still in the process of shopping the most favorable venue for their preferred solutions. As our argument suggests, this puts the German AI policy subsystem closer to the nascent than to the mature pole of the spectrum.

### Tentative support for agenda convergence over time

**Fig. 3:**
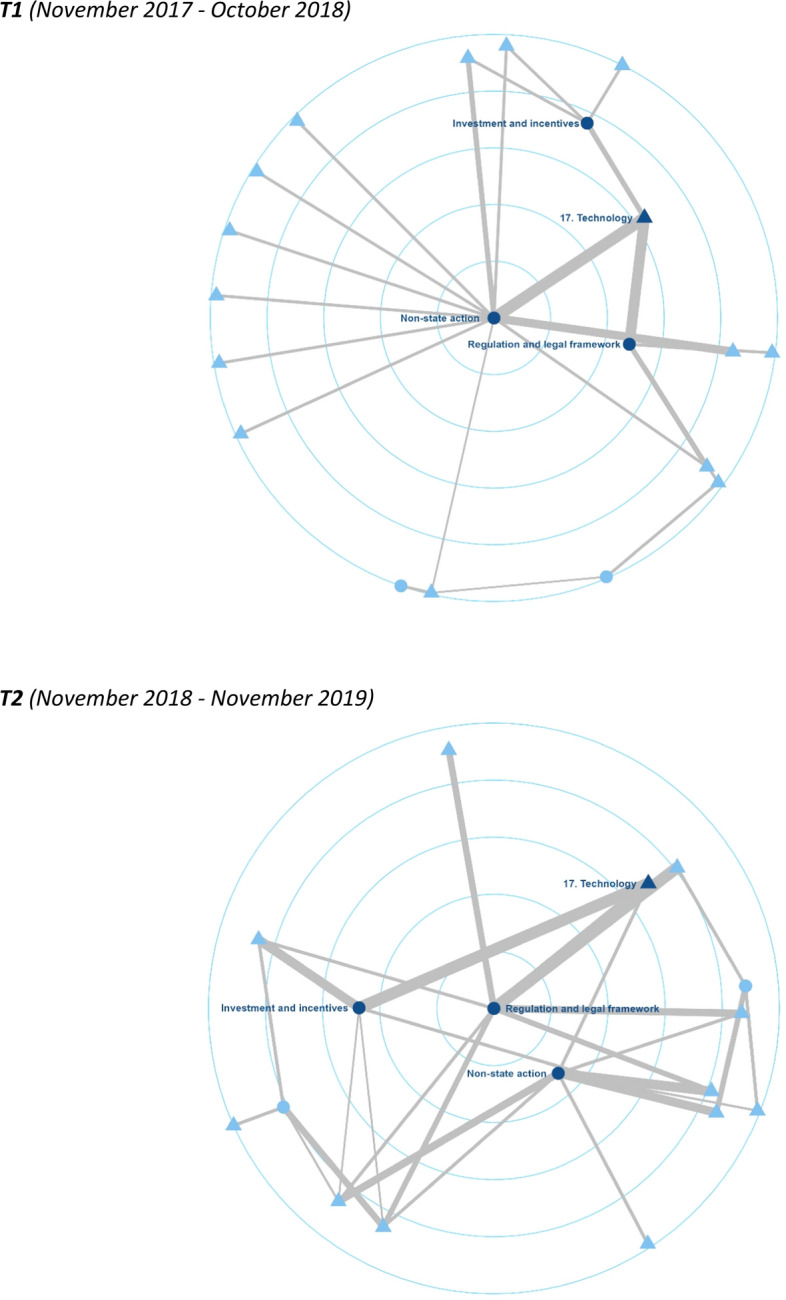
*Temporal networks in the media (centrality layout)*. T1 (November 2017—October 2018). T2 (November 2018—November 2019). Two-mode networks (from top to bottom: time point 1, time point 2): Nodes: Policy issues are depicted as triangles, while policy instruments are depicted as circles. The dark blue color highlights the five issue nodes and the two instrument nodes with the highest degree centralities. Edges: Their width denotes the strength of each edge

Figure 3 tracks the development of the policy agenda in the print media over the periods between November 2017 and October 2018 and between November 2018 and November 2019 (Figure 5 in the Online Appendix shows the two-mode networks of the parliamentary debates). We pre-processed the data in R (version 4.2.2, R Core Team, 2020) and visualized the networks with igraph (Csardi & Nepusz, 2006). The longitudinal comparison of the networks reveals changes in both the parliamentary and the print media debates, although the changes are more pronounced in the latter than in the former. In the parliamentary networks, there was a clear increase in the number of policy issues on the agenda between the first and the second period. Additionally, the policy instrument of regulation and legal framework became more important in the second period. The print media networks appear to be marked by a notable increase in the importance of the policy instruments of regulation and legal framework and investment and incentives during the second period, at the expense of non-state action.

Together with the cross-sectional comparison between the two venues, these changes suggest that the agendas of the parliament and the print media converged over time (between November 2018 and November 2019). The higher similarity appears to be driven mainly by the increased importance of the regulatory policy instrument in the two venues and of the investment and incentives tool in the second venue. The increased importance of regulation and legal framework in the two venues can likely be traced back to the adoption of the German national strategy on AI in autumn 2018. Overall, the findings from the temporal comparisons show tentative evidence of a more mature German AI policy subsystem in the second period, with agendas converging over time and across venues and with one instrument playing the key role of the “glue” behind this development. Additionally, they demonstrate that our cross-sectional approach of comparing different venues can be usefully combined with a longitudinal perspective.

Taken together, our analysis of the policy debates that took place in the print media, the parliament, and in the government consultation presents a nuanced picture of the policy agenda for AI in Germany. We find support for some overlap in policy issue priorities across the three venues. However, the links between policy issues and instruments only show limited similarity across venues. Our temporal comparisons offer some tentative evidence of increasing similarity and agenda convergence over time. In other words, actors in Germany’s AI policy subsystem seem to have begun referring to the same policy problems (issues). However, they do not reference the exact same solutions (instruments) yet. Instead, policy actors advocate for different policy mixes across the three venues. In line with what we would expect of an emerging issue area around a relatively novel topic, such as AI, Germany’s AI policy subsystem appears to be more nascent than mature.

## Discussion

We should treat the implications of our illustrative case study with caution. Our findings could be corroborated by testing the empirical approach employed in this article on a range of other subsystems, including more mature ones. Comparing Germany’s nascent AI policy subsystem to a potentially more mature AI policy subsystem, such as its U.S. counterpart, might be an appropriate next step. Such a comparison could also help evaluate different real-world policy subsystems’ levels of nascency and maturity and place them along the continuum from quite nascent to very mature. Furthermore, longitudinal comparisons of (debate) networks across venues could be a useful way of exploring the theoretical arguments outlined in this study. They could, for example, lead to insights into the relationship between temporal development and maturity. Additionally, future research could empirically test the relationship between the development of subnational, national, and supranational policy subsystems and vertical venue-shopping.

Moreover, while the two-mode network approach allows us to analyze the relationship between policy problems and policy solutions on a subsystem’s agenda, we do not account for the relationships between nodes of the same type, i.e., among issues or among instruments. Future research could incorporate the one-mode projections of the two-mode networks to, for example, analyze which policy solutions connect different problems. Additionally, different venues are more active during different phases of the policy cycle, which generates concern about their comparability. While the government consultation provided an interesting snapshot, especially at an early stage of the policymaking process, it could not be included in longitudinal analyses. There also is a key difference between an institutional ‘venue’, which produces binding decisions, such as parliaments, governments, agencies, and courts and an ‘arena’ of public debate, such as the media, where no binding decisions are made, but which covers – and influences – the decision-making process in institutional venues. Venues are also interdependent, which our current analysis does not account for. Spill-over effects, if present, could be an important indicator of agenda convergence and thus identify a more mature policy subsystem.

Scholars could adopt a process-tracing approach, follow the venues in which their actors are active (in line with the idea of venue-shopping), and connect them to the key binding decisions being made. Alternatively, scholars could focus on the venues that are a priori most relevant to a political system (e.g., parliament, government, court, direct democracy), which vary across countries.

Finally, whether AI will turn into a policy subsystem similar to health, labor, or energy remains an empirically open question. Recent scholarship casts doubt on this assumption because AI is widely applicable across sectors (Büthe et al., 2022). Based on the results from our empirical analysis of the period between 2017 and 2019, both options (an AI policy subsystem and sectoral AI policies) certainly remain a possibility for German AI policymaking. Nevertheless, the upcoming EU AI Act (Justo-Hanani, 2022) could influence the emergences of national-level AI policy subsystems, especially in the realm of high-risk applications.

## Conclusion

With this article, we aim to make a theoretical and an empirical contribution to the scholarship on nascent policy subsystems. Despite recent advances in the research on nascent policy subsystems, a subsystem’s nascency is more often assumed (because of its young age) than empirically ascertained. This article contributes to filling this gap by mobilizing arguments on agenda-setting and venue shopping to theorize how policy agendas in emerging issue areas, i.e., more nascent policy subsystems, differ from more established, mature subsystems’ agendas. By leveraging arguments from the PET instead of the ACF, which currently dominates the studies on nascent policy subsystems, we open the door to further cross fertilization between research on nascent policy subsystems and scholarship on policy process theories.

Specifically, we develop two expectations that help us describe the dynamics of agenda setting in nascent policy subsystems: (1) In nascent policy subsystems, actors emphasize diverse policy issues and instruments across different venues, in contrast to the uniformity we find in mature policy subsystems. (2) As a policy subsystem becomes more mature, the diversity in policy issues and instruments that actors emphasize declines over time. Furthermore, we argue that discourse network analysis (Leifeld, 2017) is a suitable method to empirically examine nascent policy subsystems.

We illustrate our argument empirically using textual data from policy debates on AI in Germany. Our findings suggest that during the studied period (2017 to 2019), policy actors started to refer to a common set of policy problems related to AI. However, despite some convergence in the media and parliamentary venues over time, the debated policy solutions continued being diverse and subject to intense venue-shopping. The emerging area of German AI policymaking thus comes closer to the nascent pole of the spectrum, in line with what we would expect of such a novel topic for policymaking.

More broadly, nascent policy subsystems are of interest to scholars who analyze the policy process in general. Nascency, identified here by the lack of a clearly defined policy agenda and thus, by extension, characterized by diverging and competing policy mixes across venues, could very well have consequences for the effectiveness of the subsystem’s policy output, as well as for the State’s capacity to manage new problems, such as those arising from disruptive technological innovations or major crises (like a pandemic). In a world of policy accumulation and complexity (Adam et al., 2018), understanding policy subsystem development could have meaningful consequences: Whether new issues form their own subsystem or get integrated into existing subsystems could, for example, play a crucial role in improving our understanding of policy success.

Future research on policy theories could extend the concept of nascent policy subsystems to other approaches in policy studies. Our article makes it possible to connect nascent policy subsystems to the Multiple Stream Framework (Kingdon 2011), for example, by connecting policy issues to the problem stream, instruments to the policy stream, and debates to the political stream. In a similar way, identifying “policy brokers,” who are supposed to set a common agenda, as a compromise between two or more competing coalitions would also bring agency back into the proposed approach (more explicitly than our empirical analysis did). Furthermore, the approach suggested in this article could help bring a network- and actor-oriented perspective to the scholarship on policy integration, which deals with the integration of new policy problems into existing subsystems, among other topics (Trein et al. 2021; Cejudo & Trein, 2023). Additional scholarship could also connect nascent policy subsystems to the Narrative Policy Framework in an effort to understand the development of policy stories in emerging policy subsystems (Jones et al., 2023). In this way, nascent policy subsystems could become part of the study of various theories of the policy process.

### Supplementary Information

Below is the link to the electronic supplementary material.

Supplementary file1 (PDF 390 KB)

## Data Availability

The data that support the findings of this study are available from the corresponding authors upon request.

## Footnotes

1 E.g., health, transportation, labor.

2 E.g., regulations, financial incentives, and information tools.

3 E.g., consultations with interest groups, media and parliamentary debates.

4 See, for example, Angela Merkel's speeches in the Bundestag on March 21^st^ 2018 and March 21^st^ 2019.

5 Federal Government of Germany [https://www.ki-strategie-deutschland.de/home.html], accessed in March 2020.
