# Supplementary material for: Agenda-setting in nascent policy subsystems: issue and instrument priorities across venues
Source: Policy Sci. 2023 Oct 21;56(4):633–55. doi: 10.1007/s11077-023-09514-5 (PMC10638171; doi:10.1007/s11077-023-09514-5)
Supplement: Supplementary file 1 — Supplementary file1 (PDF 390 KB) [file 11077_2023_9514_MOESM1_ESM.pdf]

Online Appendix to the paper entitled:

Agenda-setting in nascent policy subsystems: Issue and instrument priorities across venues

Table 1: Actors present in the different venues

**Actors**			
Type	Parliament *(6 unique actors)*	Newspapers *(71 unique actors)*	Consultation *(87 unique actors)*
Political parties *(All parties in the first chamber of the federal parliament)*	6	7 *(Including the Pirate party not represented in parliament)*	1
Interest groups *(Business and public interest groups)*		9	35
Private firms	-	26	27
Governmental actors *(Different ministries and government organizations)*		15	3
Scientific actors *(Actors related to research and Universities)*	-	12	21
International *(e.g. EU institutions and international NGOs)*	-	2	-

Table 2: Coding examples

Venue	Actor	Statement (translated)	Statement (original)	Coding decision
Policy issues				
Media	Industrial organization	describing the EU AI regulation as “an important step for AI made in Europe”, but warning to “treat all applications the same way”	“bezeichnete die EU Leitlinien als wichtigen Schritt für Künstliche Intelligenz “made in Europe”, warnte aber zugleich davor alle Anwendungen über einen Kamm zu scheren”	Technology
Government consultation	University	“it should be taken into account that the early setting of standards can have a high significance for competitiveness”	“insbesondere sollte berücksichtigt werden, das das frühzeitige Setzen von Standards eine hohe wettbewerbliche Bedeutung besitzen kann”	Domestic commerce
Parliament	Member of Liberals	“Why is there no center for digital vocational training?”	“warum gibt es kein Zentrum für digitale berufliche Bildung?”	Education
Government consultation	Employer association	“one key in this transformation process will be (...) the further training of employees.”	“Ein Schlüssel in diesem Transformationsprozess wird (...) die Weiterbildung .” der Mitarbeiterinnen und Mitarbeiter haben”	Labor
Parliament	Member of Christian Democrats	“We believe that the structures with the Minister of State in the Federal Chancellery, with the Digital Cabinet or the State Secretary Committee, which coordinates, are absolutely the right way to go”	“Die Strukturen mit der Staatsministerin im Bundeskanzleramt, mit dem Digitalkabinett bzw. dem Staatssekretärsausschuss, der koordiniert, halten wir für den absolut richtigen Weg”	Government operations
Media	University expert	pointing out that AI systems can have “measurement errors based on familiar biases”	“sich aufgrund vertrauter Vorurteile vermessen”	Civil rights
Media	Physicians’ professional association	indirectly cited in the media stating about algorithms in diagnostic apps that it is “important that there is always a	“wichtig sei, dass am Ende dieser Angebote immer ein Vertragsarzt stehe”	Health

		contracted physician at the end of these offers”		
Policy instruments				
Media	Firm	“risks should not be ignored, but we must not allow ourselves to be led by fear, but rather take advantage of design opportunities”	“Risiken sollten nicht ignoriert werden, aber wir dürfen uns nicht von Angst leiten lassen, sondern Gestaltungschancen nutzen”	Non-state action
Media	University expert	“we need an awareness that good regulation is not an obstacle to research and business, but that this is precisely what can create confidence in AI systems”	“Wir brauchen ein Bewusstsein dafür, dass gute Regulierung kein Hindernis für Forschung und Wirtschaft ist, sondern dass gerade dadurch Vertrauen in KI-Systeme geschaffen werden kann”	Regulation and legal framework
Government consultation	Health insurer	“for the successful use of AI technologies in healthcare, it is necessary (...) above all to overcome emotional fears and acceptance problems”	“Für den erfolgreichen Einsatz von KI Technologien im Gesundheitswesen ist es notwendig (…) vor allem auch emotionale Ängste und Akzeptanzprobleme zu überwinden”	Information and education
Government consultation	Trade union	demanded “mandatory labeling of chatbots and system-generated messages/media content”	“Kennzeichnungspflicht für Chatbots sowie von Systemen generierten Nachrichten/Medieninhalten”	Regulation and legal framework
Parliament	Member of the Liberals	"But why is there not an investment in an agency for radical innovation, as in Switzerland, as in the U.S.?"	“Aber warum wird nicht wie in der Schweiz, wie in den USA in eine Agentur für radikale Innovationen investiert?”	Investments and incentives
Parliament	Member of the Greens	refers to the parliamentary enquete commission on AI, saying “it is good that we are setting up this commission of inquiry here today”	“es ist gut dass wir diese Enquete Kommission heute hier einsetzen”	Information and education

Fig. 4: Policy issue – policy instrument networks across policy venues (grouped layout)

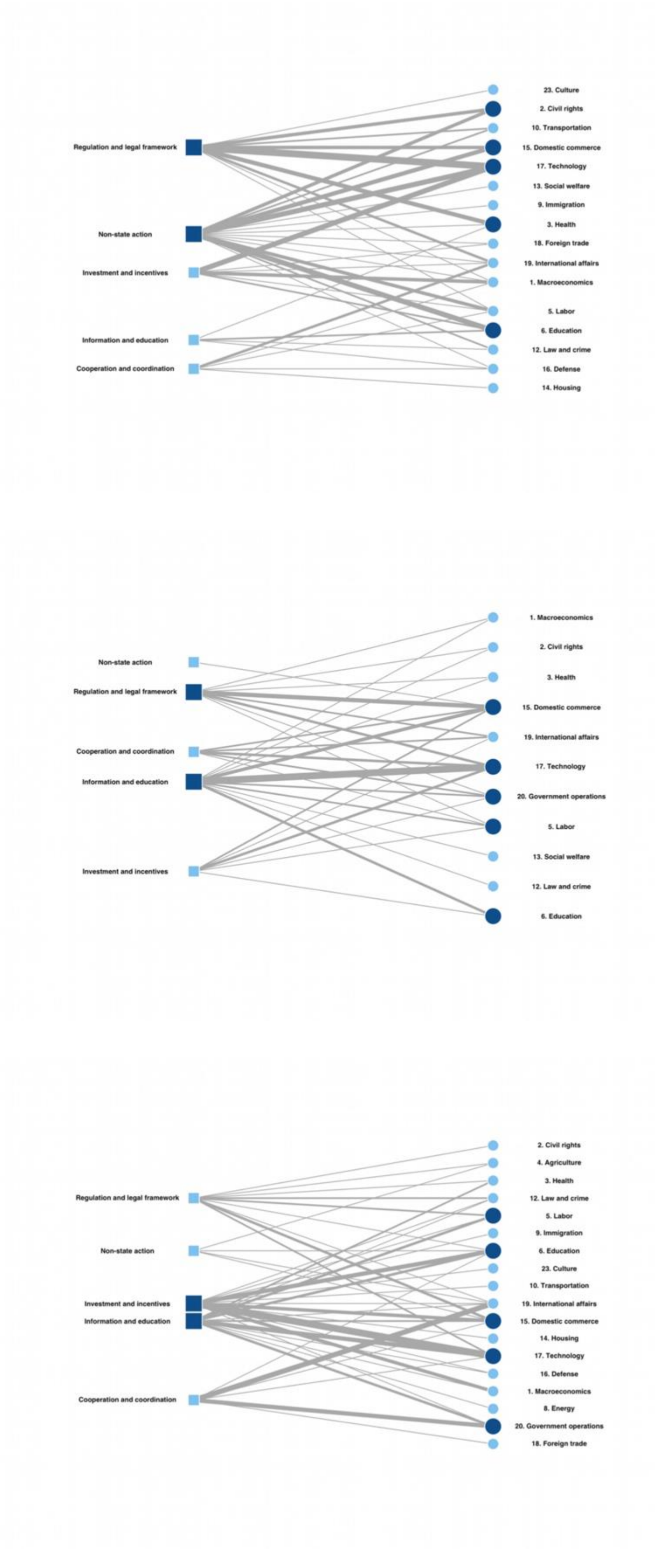

Two-mode networks. From top to bottom: media, consultation, parliament. Nodes: A set of instrument nodes (squares) on the left-hand side and a set of CAP nodes (circles) on the right-hand side. The dark blue color and the nodes' size highlight the two nodes with the highest degree centralities on the left (instruments) and the five nodes with the highest degree centralities on the right (CAP). Edges: Greater width denotes stronger edges.

Fig. 5: Temporal networks parliament

T1

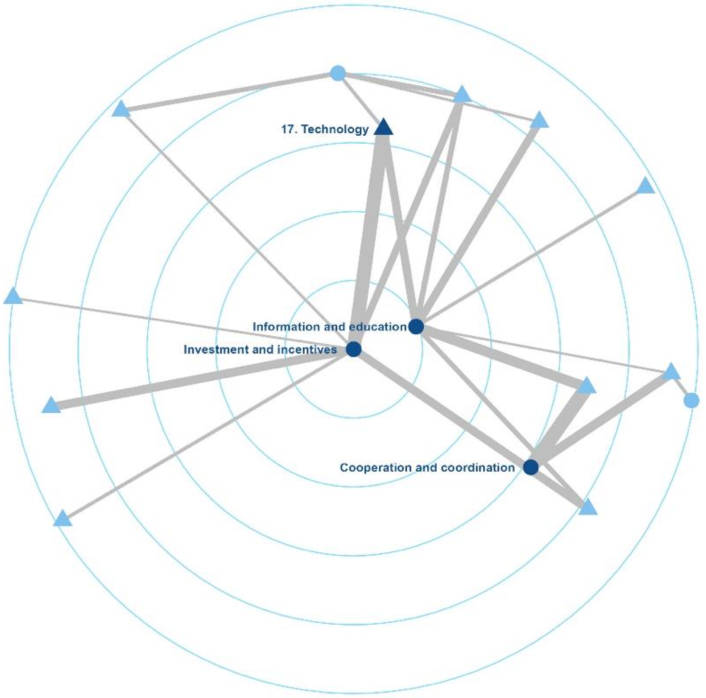

T2

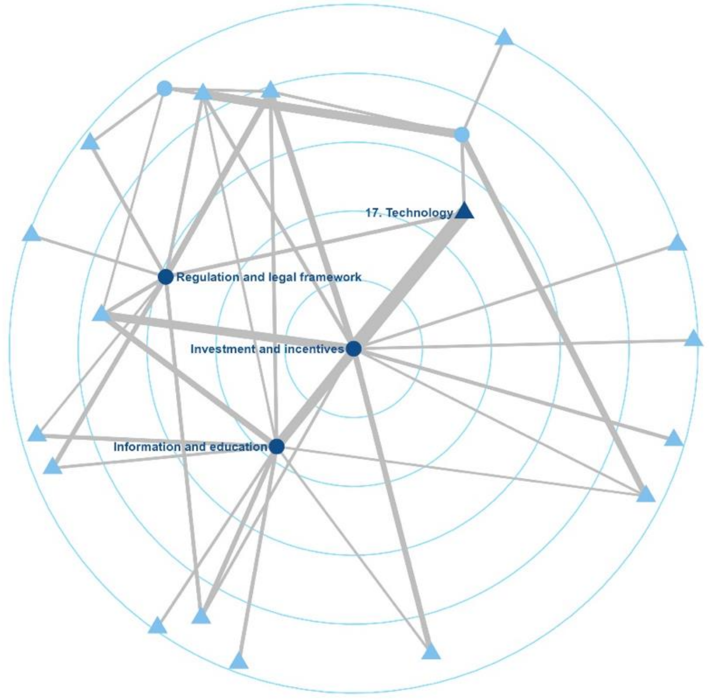

Two-mode networks (centrality layout). From top to bottom: time point 1, time point 2. Nodes: Policy issues are depicted as triangles, while policy instruments are depicted as circles. The dark blue color highlights the five issue nodes and the two instrument nodes with the hi ghest degree centralities. Edges: Width denotes each edge's strength.
